# Waste-to-Fuels: Pyrolysis of Low-Density Polyethylene Waste in the Presence of H-ZSM-11

**DOI:** 10.3390/polym13081198

**Published:** 2021-04-07

**Authors:** Nahyeon Lee, Junghee Joo, Kun-Yi Andrew Lin, Jechan Lee

**Affiliations:** 1 Department of Energy Systems Research, Ajou University, 206 World Cup-Ro, Suwon 16499, Korea; skgus@ajou.ac.kr; 2 Department of Environmental and Safety Engineering, Ajou University, 206 World Cup-Ro, Suwon 16499, Korea; jurno@ajou.ac.kr; 3 Development Center of Sustainable Agriculture, Department of Environmental Engineering & Innovation, National Chung Hsing University, 250 Kuo-Kuang Road, Taichung 402, Taiwan

**Keywords:** waste-to-energy, thermochemical process, hydrocarbon, zeolite, plastic upcycling

## Abstract

Herein, the pyrolysis of low-density polyethylene (LDPE) scrap in the presence of a H-ZSM-11 zeolite was conducted as an effort to valorize plastic waste to fuel-range chemicals. The LDPE-derived pyrolytic gas was composed of low-molecular-weight aliphatic hydrocarbons (e.g., methane, ethane, propane, ethylene, and propylene) and hydrogen. An increase in pyrolysis temperature led to increasing the gaseous hydrocarbon yields for the pyrolysis of LDPE. Using the H-ZSM-11 catalyst in the pyrolysis of LDPE greatly enhanced the content of propylene in the pyrolytic gas because of promoted dehydrogenation of propane formed during the pyrolysis. Apart from the light aliphatic hydrocarbons, jet fuel-, diesel-, and motor oil-range hydrocarbons were found in the pyrolytic liquid for the non-catalytic and catalytic pyrolysis. The change in pyrolysis temperature for the catalytic pyrolysis affected the hydrocarbon compositions of the pyrolytic liquid more materially than for the non-catalytic pyrolysis. This study experimentally showed that H-ZSM-11 can be effective at producing fuel-range hydrocarbons from LDPE waste through pyrolysis. The results would contribute to the development of waste valorization process via plastic upcycling.

## 1. Introduction

Disposal of plastics is an important contemporary problem. Even though waste plastics have been commonly landfilled, landfilling the waste is no longer acceptable because plastics have low weight-to-volume ratios causing serious environmental problems. Efforts to address the concerns about plastic waste disposal have resulted in manufacturing photo- and biodegradable plastics. However, it generally takes years to degrade these plastics, and stabilizers that are harmful to the environment are used during the manufacture of the degradable plastics. Incineration of waste plastics is another option to dispose of them. Nevertheless, incineration causes the loss of chemical content and energy of the plastics (i.e., carbon) with the emission of harmful and toxic byproducts into the atmosphere. Recycling is a more preferred way to treat plastic waste because it has less environmental impacts than landfilling and incineration. However, recycling of plastics is technically limited due to the difficulty in separation. For instance, the separation of plastics from municipal solid wastes (MSWs) is done mostly by hand by householders before collection and at recycling facilities [1]. Therefore, the employment of plastic waste as the feedstock for fuels and chemicals (i.e., upcycling of plastic waste) is more attractive than the aforementioned methods for treating waste plastics (e.g., landfilling, manufacturing degradable plastics, incineration, and recycling).

Low-density polyethylene (LDPE) is one of the most widely used plastics in various applications because of its excellent physico-chemical properties. For example, LDPE has low toxicity, light mass, mechanical durability, superior chemical resistance, and excellent electrical insulation with ease of molding and processing [2,3]. Therefore, the processes for converting waste plastics into fuels and value-added chemicals (e.g., lubricants) [4,5] have attracted much interest. Among the plastics, LDPE is suitable for producing fuel-ranged hydrocarbons (i.e., oil) because it has a high effective H/C ratio (~2) and molecular chain structure [6].

Thermochemical conversion of carbonaceous substances (e.g., polymers and biomass), such as pyrolysis and liquefaction, has been recognized as a promising option to recover energy in the form of liquid (e.g., bio-oil). Between the two, pyrolysis is more preferable to generating oil from plastics than liquefaction because the liquefaction process requires a wet pretreatment step prior to liquefying plastics that is expensive and generates extra waste streams [7,8]. Pyrolysis not only treats feedstocks that are relatively contaminated but also reusable materials (e.g., metals) can be recovered before fuel production processing actually begins [9]. In addition to these advantages, pyrolysis is a versatile process suitable for scale-up to produce a range of chemical products. As an example, polyolefin feedstocks could be transformed into waxes, aromatics, and olefin gases [10]. Furthermore, it was found by a comparative life cycle analysis that pyrolysis is one of the most environmentally benign routes to transform plastic wastes into oil [11].

Pyrolysis of LDPE has been used to produce a range of hydrocarbons mainly with various zeolites. For example, Mordi et al. used H-theta-l, H-mordenite, and H-ZSM-5 for pyrolysis of LDPE to convert it into hydrocarbons [12]. They concluded that pyrolysis of LDPE catalyzed by H-ZSM-5 resulted in the product containing more aromatic compounds than that catalyzed by H-theta-l and H-mordenite. In a previous study, LDPE was pyrolyzed in a fluidized bed reactor, indicating that the chemical composition of the LDPE-derived oil was associated with pyrolysis temperature [13]. H-beta and H-ZSM-5 catalysts were also used to thermally decompose LDPE [14]. Among various kinds of zeolite catalysts, H-ZSM-11 has similar pore dimensions to H-ZSM-5 with straight intersecting channels and elliptical pore size of 0.53 × 0.54 nm [15,16,17]. Its diffusion resistance is lower than H-ZSM-5 [18]; thereby, H-ZSM-11 showed a higher catalytic activity for catalytic cracking [19], aromatization, isomerization [20], and methanol-to-hydrocarbons [21] than HZSM-5. Nevertheless, catalytic pyrolysis of LDPE with H-ZSM-11 has rarely been investigated, unlike H-ZSM-5.

In this respect, this study has attempted to evaluate the effectiveness of H-ZSM-11-catalyzed pyrolysis for valorization of LDPE waste to fuel-range chemicals. To the best of the authors’ knowledge, this is the first study applying the H-ZSM-11 catalyst to upcycling of LDPE waste via pyrolysis process. The effects of pyrolysis conditions (e.g., temperature and inert gas flow rate) were studied by qualitatively analyzing pyrolytic products produced to evaluate the effectiveness of H-ZSM-11 catalyst in pyrolysis of LDPE. This study will help develop a waste-to-energy process to upcycle everyday waste such as LDPE.

## 2. Materials and Methods

### 2.1. Materials

LDPE scrap was collected from a local waste recycling center located in Suwon, Gyeonggi province, Republic of Korea. A ZSM-11 zeolite was supplied from Vision Chemicals (Castleford, UK). The H-form of the ZMS-11 was made by its calcination at 550 °C for 3 h.

Thermogravimetric analysis (TGA) of the LDPE sample was conducted using an STA 409 PC thermal analyzer manufactured by NETZSCH (Selb, Germany). The sample (10 mg) was heated from 25 to 800 °C (ramping rate: 10 °C min^−1^) under N_2_ environment. The flow rate of purge gas (N_2_) and protective gas (N_2_) was 40 mL min^−1^ and 20 mL min^−1^, respectively.

### 2.2. Catalyst Characterization

Bulk crystalline structure of the H-ZSM-11 catalyst was seen by X-ray diffraction (XRD) technique. An Ultima IV X-ray diffractometer manufactured by Rigaku (Tokyo, Japan) was used, operated with Cu Kα radiation (λ = 0.154 nm). Inorganic crystal structure database (ISCD) was referred to identify crystalline phase of XRD peaks.

Physisorption of the H-ZSM-11 catalyst was performed using an ASAP 2020 accelerated surface area and porosimetry system manufactured by Micromeritics (Norcross, GA, USA). Two different gases such as N_2_ (−196 °C) and Ar (−185 °C) were applied. Prior to physisorption, the catalyst was degassed at 150 °C in a vacuum for 4 h (N_2_-physisorption) or 12 h (Ar-physisorption).

Temperature-programmed desorption of NH_3_ (NH_3_-TPD) was conducted to measure surface acidity of the H-ZSM-11 catalyst using an Autochem II 2920 chemisorption analyzer manufactured by Micromeritics (Norcross, GA, USA). The catalyst was degassed in He flow of 50 mL min^−1^ at 500 °C for 1 h prior to NH_3_-TPD. After degassing, NH_3_ (15 vol.% in He) was adsorbed onto the catalyst at 150 °C for 0.5 h, followed by purging in He flow of 50 mL min^−1^ for 1.2 h. After purging, the adsorbed NH_3_ was desorbed from 150 to 500 °C with a heating rate of 5 °C min^−1^. The amount of desorbed NH_3_ was measured by a thermal conductivity detector.

To measure the amount of coke deposited on the catalyst, TGA of used catalyst was conducted from 30 to 900 °C with a heating rate of 10 °C min^−1^ in air flow of 100 mL min^−1^. A STA449 F3 thermal analyzer manufactured by NETZSCH (Selb, Bavaria, Germany) was used. The amount of coke deposition was calculated based on weight loss of the used catalyst.

### 2.3. Pyrolysis Experimental Setup

A pyrolyzer was built, composed of a split-hinge tube furnace manufactured by HANTECH (Gunpo, Gyeonggi, Korea), a mass flow controller (MFC) manufactured by KOFLOC (Kyoto, Japan), and a two-stage condenser (−1 °C and −40 °C). A quartz tube loading LDPE waste (1 g) was put in the furnace, and the feedstock is located in the center of the furnace heating zone. A temperature controller was equipped with the furnace to control pyrolysis temperature with a heating rate of 10 °C min^−1^. Ultra-high purity N_2_ gas was supplied through the MFC. For catalytic pyrolysis, the H-ZSM-11 catalyst (10 mg, equivalent to 1 wt.% of the feedstock) was mixed with the feedstock.

### 2.4. Product Analysis

Condensable species were collected in the two-stage condenser. The collected condensables were analyzed by a gas chromatograph–mass spectrometry (GC–MS) manufactured by Agilent Technologies (Santa Clara, CA, USA). For quantification of condensable compounds, an internal standard (5-methylfurfural; 5 μg mL^−1^) was used. A micro-GC manufactured by INFICON (Bad Ragaz, Switzerland) was employed to analyze non-condensable gases, which was directly connected to the pyrolyzer outlet. Specification, column information, and analytical conditions used for the GC–MS analysis, and micro-GC analyses are provided in Tables S1 and S2, respectively.

## 3. Results and Discussion

### 3.1. Characterization of Feedstock and Catalyst

**Figure 1:**
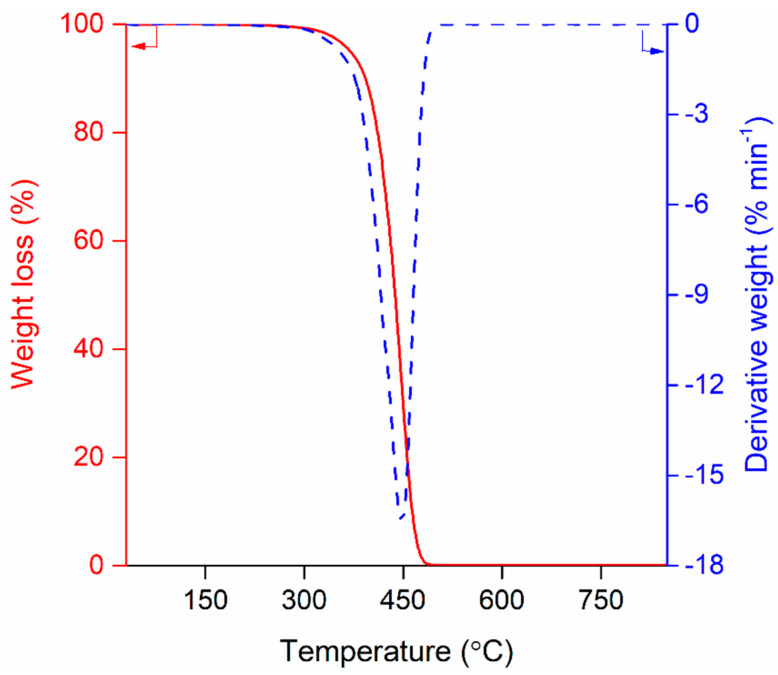
Thermogravimetric analysis (TGA) and derivative weight profiles of the low-density polyethylene (LDPE) sample used as the feedstock in this study.

The properties of the LDPE sample used as the feedstock in this study are given in Table 1. As the TGA profile (Figure 1) indicates, the LDPE sample showed a single weight loss upon thermal degradation from 300 °C to 500 °C in N_2_ atmosphere. It should be notable to mention that the thermal decomposition of the LDPE sample observed in the TGA experiment only reflects its physical degradation. Pyrolysis temperature has effects on compositions of the chemical compounds made during pyrolysis of LDPE in a far more susceptive manner than seen in the TGA profile (Figure 1) [22,23].

The changes in characteristics of the fresh H-ZSM-11 catalyst and the H-ZSM-11 catalyst after the pyrolysis of LDPE were measured. Structural characteristics of the two were measured by XRD analysis, as seen in Figure S1. A marked change in crystalline structure of the H-ZSM-11 catalyst was not observed except for a slight shift in baseline near around 20° and 30°. This indicates deposition of amorphous coke onto the catalyst during the pyrolysis of LDPE.

Table S3 lists physicochemical properties of the fresh H-ZSM-11 and the H-ZSM-11 after the pyrolysis of LDPE. The H-ZSM-11 catalyst lost more than 90% both of its BET surface area and of external surface area during the pyrolysis. It was observed that the volume of micropores of the H-ZSM-11 considerably decreased after the pyrolysis with no demolition of its crystallite structure (Figure S1). This means that the decrease in the volume of micropores is ascribed to the formation of coke onto the catalyst. Coke of 8.9 wt.% was determined by TGA of the H-ZSM-11 catalyst after pyrolysis (Table S3). The coke deposited onto the catalyst surface reduced the surface acidity of the H-ZSM-11 catalyst [24,25], as shown in Figure S2 and Table S3. As seen in Figure S2, the fresh H-ZSM-11 catalyst shows two NH_3_-TPD peaks centered at 200 °C and 350 °C, which are associated with weakly adsorbed NH_3_ and strongly adsorbed NH_3_, respectively [26]. However, the amount of surface acid sites of the H-ZSM-11 catalyst decreased by 40% after the pyrolysis of LDPE. This is most likely because coke blocks external surface and/or micropores of the catalyst during the pyrolysis [27].

### 3.2. Pyrolysis of LDPE without and with H-ZSM-11 Catalyst

In order to investigate the effect of the H-ZSM-11 catalyst, both non-catalytic pyrolysis and catalytic pyrolysis were conducted. Accordingly, the yields and compositions of pyrolytic products produced from the non-catalytic and catalytic pyrolysis of LDPE were compared. An inert atmosphere during the pyrolysis was maintained by flowing N_2_ gas. The effect of N_2_ flow rate was investigated first by changing the flow rate from 100 mL min^−1^ to 300 mL min^−1^, and no change in the yields and compositions of pyrolytic products was found. Therefore, 100 mL min^−1^ was chosen as the flow rate of N_2_ gas for further experiments.

**Figure 2:**
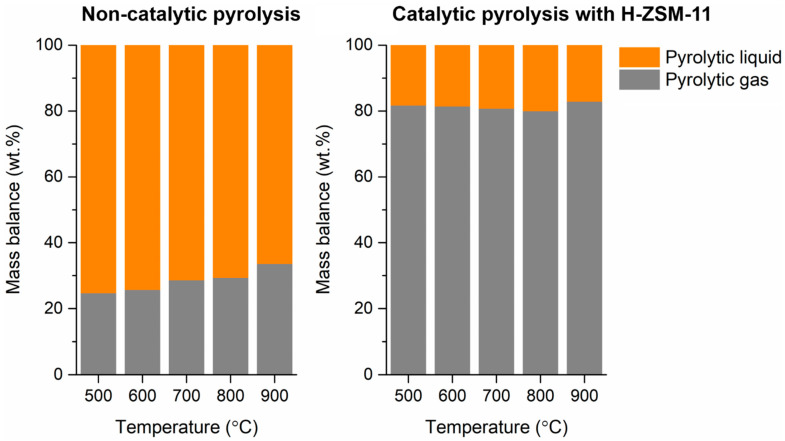
Overall mass balances of pyrolytic products produced via the pyrolysis of LDPE without and with the H-ZSM-11 catalyst as functions of pyrolysis temperature. Average values of triplicates (*n* = 3) are reported and standard deviations of the average values of around 3–6%.

Figure 2 shows overall mass balances of the pyrolytic products produced from the LDPE sample without and with the H-ZSM-11 catalyst at different pyrolysis temperatures. On a per mass basis, the most produced product was pyrolytic liquid at all tested temperatures from 500 °C to 900 °C. No solid product was made at any of the temperatures, either for non-catalytic pyrolysis or for catalytic pyrolysis. This is an indication of the complete thermal decomposition of LDPE at temperatures higher than 500 °C (also refer Figure 1). For the non-catalytic pyrolysis of LDPE, an increase in pyrolysis temperature tended to enhance the yield of the pyrolytic gas consisting of permanent gases. Higher temperatures favor thermal cracking of pyrolytic vapor because gas phase homogeneous reactions and gas–solid phase heterogeneous reactions are promoted [28,29]. The use of the H-ZSM-11 catalyst greatly increased the yield of pyrolytic gas. For example, at 700 °C, the yield of pyrolytic gas obtained through the non-catalytic pyrolysis was 28.6 wt.%, while that obtained through the catalytic pyrolysis was 80.8 wt.%. This clearly indicates that the H-ZSM-11 catalyst expedites thermal cracking of high-molecular-weight compounds contained in the pyrolytic vapor evolved from LDPE during its pyrolysis.

**Figure 3:**
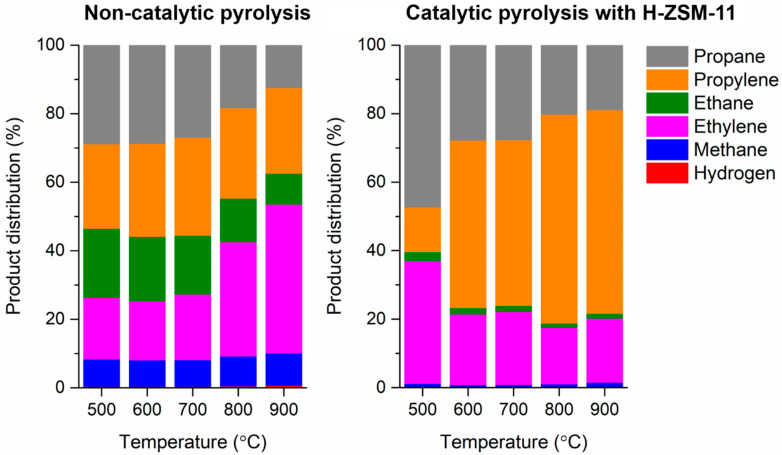
Distributions of non-condensable gases in the pyrolytic gas produced via the pyrolysis of LDPE without and with the H-ZSM-11 catalyst as functions of pyrolysis temperature. Average values of triplicates (*n* = 3) are reported and standard deviations of the average values of around 3–6%.

The pyrolytic gas is a mixture of permanent gases (i.e., non-condensable gases). Figure 3 shows distributions of permanent gases in the pyrolytic gas produced via the non-catalytic and catalytic pyrolysis of LDPE as functions of pyrolysis temperature. The permanent gases involved hydrogen and light aliphatic hydrocarbons such as methane, ethylene, ethane, propylene, and propane. For the non-catalytic pyrolysis of LDPE, the distribution of permanent gases was not affected materially by the temperature between 500 °C and 700 °C. However, at temperatures higher than 700 °C, the content of ethylene increased with a decreased content of propane. Dehydrogenation of propane leads to propylene, and disproportionation of propylene leads to ethylene [30]. Thus, this means that dehydrogenation of propane and disproportionation of propylene are expedited at temperatures higher than 700 °C. In addition to the propane dehydrogenation and propylene disproportionation, enhanced thermal depolymerization of polyethylene could make more ethylene at higher temperatures. At >700 °C, the content of ethylene increased, but the content of ethane decreased as the temperature became higher. This is most likely because hydrogenation of ethylene is hindered at >700 °C for the pyrolysis of LDPE. Dehydrogenation reactions taking place during the pyrolysis make hydrogen [31]. Thermal cracking of pyrolytic vapor produces methane [32]. The employment of the H-ZSM-11 catalyst markedly increases the content of propylene compared to the non-catalytic pyrolysis. The enhanced yield of propylene in the presence of the H-ZSM-11 could be because the catalyst promotes dehydrogenation of propane. It is known that zeolite materials catalyze dehydrogenation of alkanes to alkenes via monomolecular and protolytic pathways [33]. The results indicate that the H-ZSM-11 catalyst enhances the formation of propane and propylene for the pyrolysis of LDPE.

**Figure 4:**
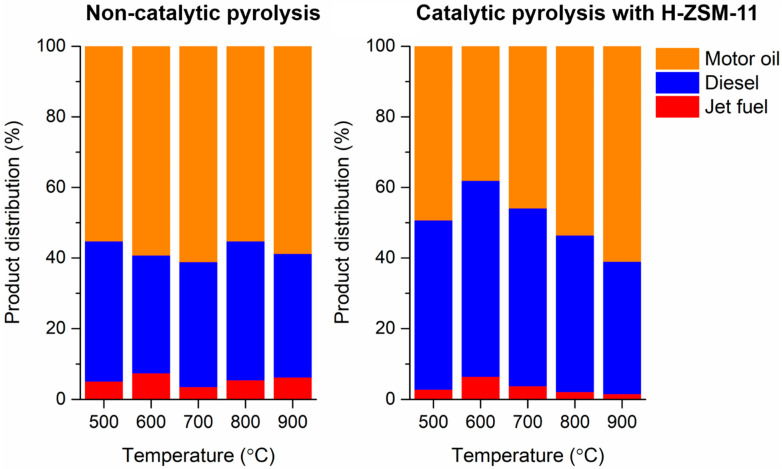
Distributions of fuel-range hydrocarbons in the pyrolytic liquid produced via the pyrolysis of LDPE without and with the H-ZSM-11 catalyst as functions of pyrolysis temperature. Average values of triplicates (*n* = 3) are reported and standard deviations of the average values of around 3–6%.

In the pyrolytic liquid produced via the non-catalytic pyrolysis of LDPE, hydrocarbons in the range of C_10_–C_44_ were identified and quantified; however, C_8_–C_35_ hydrocarbons were identified in the pyrolytic liquid produced via the catalytic pyrolysis in the presence of the H-ZSM-11, as summarized in Table 2. This shows that the H-ZSM-11 catalyst may have an effect on cracking high-molecular-weight hydrocarbons. The hydrocarbons made by the pyrolysis of LDPE could be categorized according to their carbon numbers as jet fuel-range hydrocarbons (C_5_–C_14_), diesel-range hydrocarbons (C_8_–C_21_), and motor oil-range hydrocarbons (C_18_–C_35_) [34]. Figure 4 presents distributions of fuel-range hydrocarbons in the pyrolytic liquid produced via the pyrolysis of LDPE without and with the H-ZSM-11 catalyst at different pyrolysis temperatures. For the non-catalytic pyrolysis, the product distributions were not noticeably changed by varying the pyrolysis temperature. However, the distribution of pyrolytic liquid made via the catalytic pyrolysis was more susceptible to temperature. For instance, the content of diesel decreased from 55.5% to 37.5% as the temperature increased from 600 °C to 900 °C, while the content of motor oil increased from 38.1% to 61.1%.

## 4. Conclusions

In this study, pyrolysis of a waste LDPE sample (LDPE scrap) was carried out. The variation in the flow rate of inert N_2_ gas had no influence on the yields and compositions of pyrolytic products. The non-condensable permanent gas (hydrogen, methane, ethylene, ethane, propylene, and propane) yields by the pyrolysis of LDPE increased with an increase in the pyrolysis temperature from 500 °C to 900 °C. At the tested temperatures, no pyrolytic solid remained. The employment of the H-ZSM-11 catalyst increased the pyrolytic gas yield, likely because thermal degradation of high-molecular-weight species evolved during the pyrolysis is expedited by the H-ZSM-11 catalyst. The H-ZSM-11 catalyst also increased the yield of propylene in the pyrolytic gas, attributed to dehydrogenation of propane promoted by the catalyst through monomolecular and protolytic pathways. In the pyrolytic liquid, hydrocarbons having a wide range of carbon numbers, classified as jet fuel-, diesel-, and motor oil-range hydrocarbons, were made via the pyrolysis of LDPE. It was found that the distribution of the fuel-range hydrocarbons in the pyrolytic liquid produced through the catalytic pyrolysis in the presence of the H-ZSM-11 was more susceptible to pyrolysis temperature that the non-catalytic pyrolysis. Pyrolytic products derived from plastic waste can be used as raw materials for some industrial sectors: pyrolytic gas as a source of energy required for pyrolysis process itself, and pyrolytic liquid as feedstock for high value chemicals and fuels [35]. Thus, the results of this study should contribute to developing more industrially feasible pyrolysis processes for the treatment of plastic waste.

## Figures and Tables

**Table 1:** Properties of the LDPE sample used as the feedstock in this study.

Density (g mL^−1^)	Transition Temperature (°C)	Melting Point (°C)	Melt Index (g min^−1^)
0.9	90	120	2.5

**Table 2:** Yields (%) of hydrocarbons with different carbon numbers in the pyrolytic liquid produced via the pyrolysis of LDPE without and with the H-ZSM-11 catalyst as functions of pyrolysis temperature.

	Non-Catalytic Pyrolysis					Catalytic Pyrolysis with H-ZSM-11				
Temperature (°C)	500	600	700	800	900	500	600	700	800	900
C_8_	0	0	0	0	0	0	0.11	0.09	0.04	0.15
C_9_	0	0	0	0	0	0	0.07	0	0	0
C_10_	0.03	0.14	0.07	0.03	0.15	0.10	0.34	0.16	0.00	0
C_11_	0.19	0.38	0.15	0.24	0.40	0.12	0.39	0.23	0.12	0
C_12_	0.54	0.94	0.38	0.59	0.81	0.40	0.97	0.34	0.26	0.08
C_13_	1.32	2.05	0.88	1.40	1.73	0.61	1.69	1.04	0.50	0.27
C_14_	2.96	3.88	2.02	3.11	3.12	1.53	2.79	1.88	1.16	1.00
C_15_	4.66	5.25	3.11	4.80	4.43	2.84	4.98	3.96	2.31	1.99
C_16_	6.59	6.73	4.94	6.53	5.84	4.99	6.53	5.71	4.04	3.11
C_17_	7.36	7.14	6.29	7.01	6.18	5.29	9.81	7.23	7.35	5.17
C_18_	0.08	0.30	0.18	0.20	0.15	8.99	9.44	9.73	7.96	7.45
C_19_	14.86	13.73	14.26	14.41	12.22	12.22	10.81	9.37	8.21	7.59
C_20_	0.03	0.14	0.16	0.15	0.12	7.80	8.32	8.62	7.87	6.17
C_21_	6.11	0.00	6.38	6.23	6.04	5.76	5.65	5.70	6.56	5.96
C_22_	0	0	0	0	0	0.24	0.24	0.05	0	0
C_23_	0.09	0.03	0.11	0.22	0.21	3.09	1.44	2.78	2.22	1.33
C_24_	6.70	6.47	7.06	6.99	6.18	0	0	0	0	0
C_26_	28.37	32.84	28.44	29.03	32.69	1.32	0.63	0.91	0.30	0.18
C_27_	0	0	3.93	0	0	5.93	5.32	5.76	6.06	4.40
C_28_	4.62	2.54	2.60	4.75	1.59	4.61	4.61	4.78	2.52	5.71
C_29_	1.03	2.01	2.69	1.23	4.97	0	4.32	0	0	0
C_35_	11.74	12.03	14.32	10.71	11.81	34.16	21.54	31.68	42.51	49.43
C_43_	0.88	1.19	0	0	1.38	0	0	0	0	0
C_44_	1.85	2.22	2.04	2.38	0	0	0	0	0	0

## Supplementary Materials

The supplementary materials are available online at https://www.mdpi.com/article/10.3390/polym13081198/s1, Figure S1: XRD patterns of the fresh H-ZSM-11 and the H-ZSM-11 after the pyrolysis of LDPE, Figure S2: NH_3_-TPD profiles of the fresh H-ZSM-11 and the H-ZSM-11 after the pyrolysis of LDPE, Table S1: Specification, column information, and analytical conditions for the GC–MS, Table S2: Specification, column information, and analytical conditions for the micro-GC, Table S3: Physicochemical properties of the fresh H-ZSM-11 and the H-ZSM-11 after the pyrolysis of LDPE.
