# Supplementary material for: Waste-to-Fuels: Pyrolysis of Low-Density Polyethylene Waste in the Presence of H-ZSM-11
Source: Polymers (Basel). 2021 Apr 7;13(8):1198. doi: 10.3390/polym13081198 (PMC8068035; doi:10.3390/polym13081198)
Supplement: Supplementary file 1 [file polymers-13-01198-s001.pdf]

Supporting Information

Waste-to-Fuels: Pyrolysis of Low-density Polyethylene Waste in the Presence of H-ZSM-11

Nahyeon Lee [a], Junghee Joo [b], Kun-Yi Andrew Lin [c,*], and Jechan Lee [a,b,*]

[a] *Department of Energy Systems Research, Ajou University, 206 World cup-ro, Suwon 16499, Republic of Korea*

[b] *Department of Environmental and Safety Engineering, Ajou University, 206 World cup-ro, Suwon 16499, Republic of Korea*

[c] *Department of Environmental Engineering & Innovation and Development Center of Sustainable Agriculture, National Chung Hsing University, 250 Kuo-Kuang Road, Taichung, Taiwan*

[*]Corresponding author. E-mail: linky@nchu.edu.tw (K.-Y.A.L.); jlee83@ajou.ac.kr (J.L.)

Table S1. Specification, column information, and analytical conditions for the GC–MS

Model	GC: Agilent 8890; MS: Agilent 5977B	
Column	HP-5MS Ultra Inlet column (0.25 mm × 0.25 um × 30 m)	
Oven setting	Initial temperature	50 °C (2 min)
	Ramping	8 °C min^{-1}
	Final temperature	290 °C (1 min)
	Total analysis time	33 min
Column setting	Carrier gas	Helium (≥99.999%)
	Carrier gas flow	3 mL min^{-1}
	Column flow	1 mL min^{-1}
Injector setting	Injection mode	Splitless
	Injection volume	1 μL
	Injection temperature	280 °C
MS setting	Aux temperature	300 °C
	m/z range	35~550 amu

Table S2. Specification, column information, and analytical conditions for the micro-GC

Model		INFICON Fusion Gas Analyzer	
Conditions		Module A	Module B
Column		Rt-Molsieve 5A	Rt-Q-Bond
Sample Pump setting	Sample pump mode	Continuous	Continuous
	Sample pump time	15 s	15 s
Column setting	Carrier gas	Argon (≥99.999%)	Helium (≥99.999%)
	Column pressure	20 psi	17 psi
	Initial temperature	50 °C (40 s)	50 °C (30 s)
	Ramping time	50 s	60 s
	Final temperature	100 °C (40 s)	110 °C (40 s)
	Total analysis time	130 s	130 s
Injector setting	Inject time	30 ms	30 ms
	Injector temperature	90 °C	90 °C
TCD setting	TCD temperature	70 °C	70 °C
	Data rate	50 Hz	50 Hz

Table S3. Physicochemical properties of the fresh H-ZSM-11 and the H-ZSM-11 after the pyrolysis of LDPE

Catalyst	BET surface area ($m^2\ g^{-1}$)	External surface area [a] ($m^2\ g^{-1}$)	Micropore volume [a] ($cm^3\ g^{-1}$)	Acid amount [b] ($\mu mol_{NH3}\ g^{-1}$)	Coke deposition [c] (wt.%)
Fresh	418	72	0.15	270	-
After pyrolysis	34	24	0.004	120	8.9

[a] Determined by the *t*-plot method
[b] Quantified by NH_3-TPD
[c] Determined by the TGA

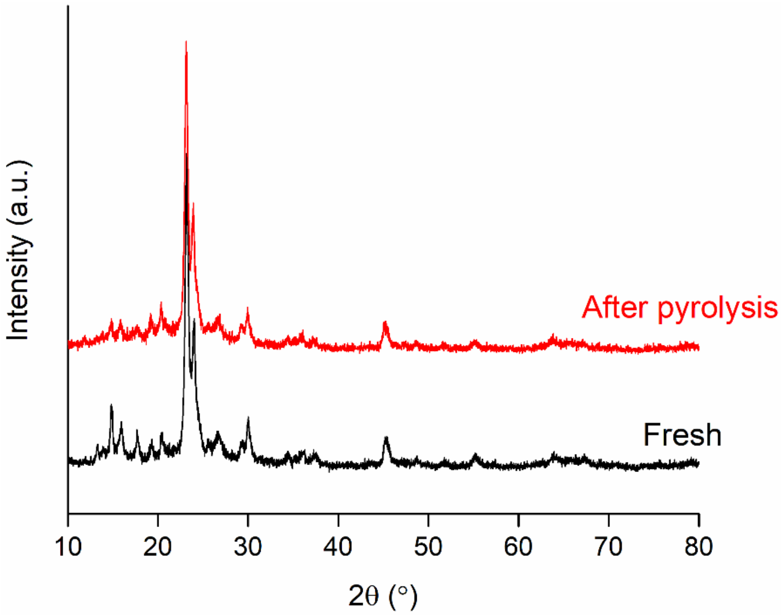

Figure S1. XRD patterns of the fresh H-ZSM-11 and the H-ZSM-11 after the pyrolysis of LDPE

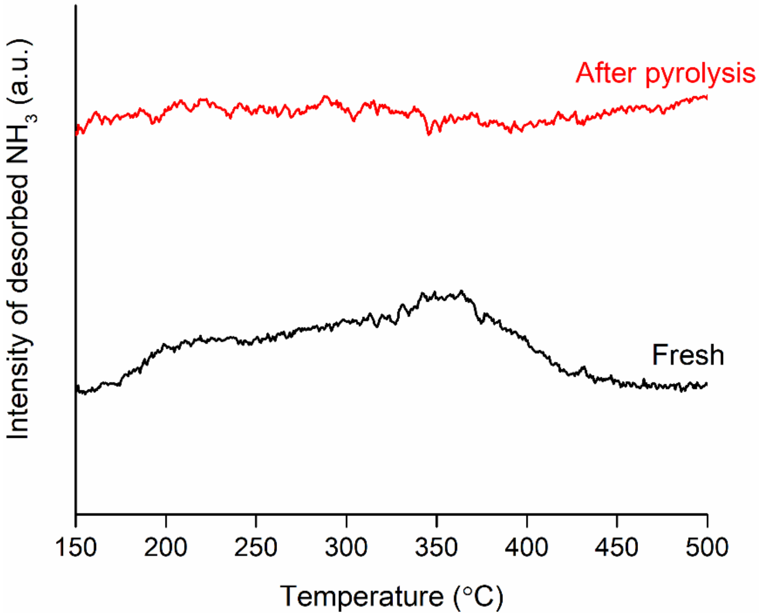

Figure S2. NH_3-TPD profiles of the fresh H-ZSM-11 and the H-ZSM-11 after the pyrolysis of LDPE.
